# Fusion of Bovine Tissue Aortic Valve Leaflets in a Patient With Left Ventricular Assist Device

**DOI:** 10.1016/j.jaccas.2022.02.009

**Published:** 2022-05-18

**Authors:** Nishi H. Patel, Cesar Guerrero-Miranda, Shelley Hall, Aldo E. Rafael, W. Morris Brown, Amarinder S. Bindra

**Affiliations:** a Wellspan Health, York Hospital, York, Pennsylvania, USA; b Center for Advanced Heart & Lung Disease, Baylor University Medical Center, Dallas, Texas, USA; c Piedmont Heart Institute, Piedmont Atlanta Hospital, Atlanta, Georgia, USA

**Keywords:** aortic valve, cardiomyopathy, left ventricle, LVAD explant, prosthetic valve stenosis, AVR, aortic valve replacement, bAVR, bioprosthetic aortic valve replacement, CT, computed tomography, LV, left ventricular, LVAD, left ventricular assist device, LVEF, left ventricular ejection fraction, TEE, transesophageal echocardiography, TTE, transthoracic echocardiography, 4D, 4-dimensional

## Abstract

Patients with both a prosthetic aortic valve and prolonged left ventricular assist device support can develop rapid deterioration of their valve prosthesis. In patients with myocardial recovery who are undergoing explantation of their ventricular assist device, preoperative and intraoperative evaluation of the valve prosthesis should be performed to ensure adequate function. (**Level of Difficulty: Advanced.**)

## Central Illustration

**Figure:**
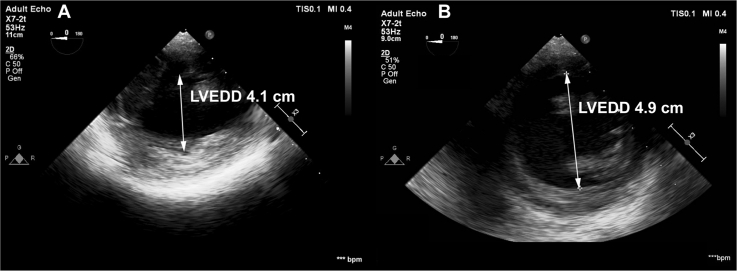

## History of Presentation

**Figure 1:**
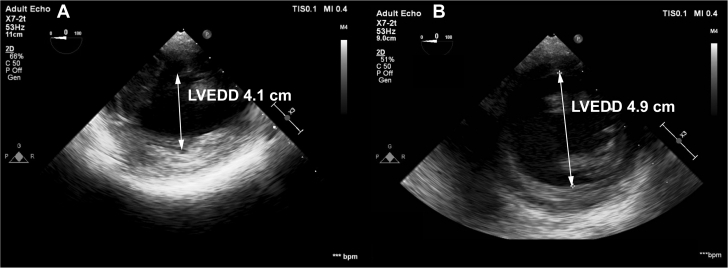
Transesophageal Echocardiography Showing Increased LVEDD **(A)** Left ventricular end-diastolic diameter (LVEDD) with the outflow graft unclamped. **(B)** LVEDD distention after outflow clamping.

A 30-year-old woman with a HeartMate 3 left ventricular (LV) assist device (LVAD) (Abbott Technologies) was admitted for LVAD explantation after showing recovery of her LV ejection fraction (LVEF) from 5% to 55%. Intraoperative clamping of the outflow graft resulted in hypotension, acute reduction of LV systolic function, and enlargement of the LV cavity, all of which resolved with unclamping of the outflow graft (Figures 1A and 1B).

**Learning Objectives**

• To understand the mechanism behind bioprosthetic aortic valve degeneration in patients with mechanical support with an LVAD.
• To identify a strategy for the preoperative functional and structural assessment of bioprosthetic aortic valves in patients undergoing LVAD explantation.

## Past Medical History

**Figure 2:**
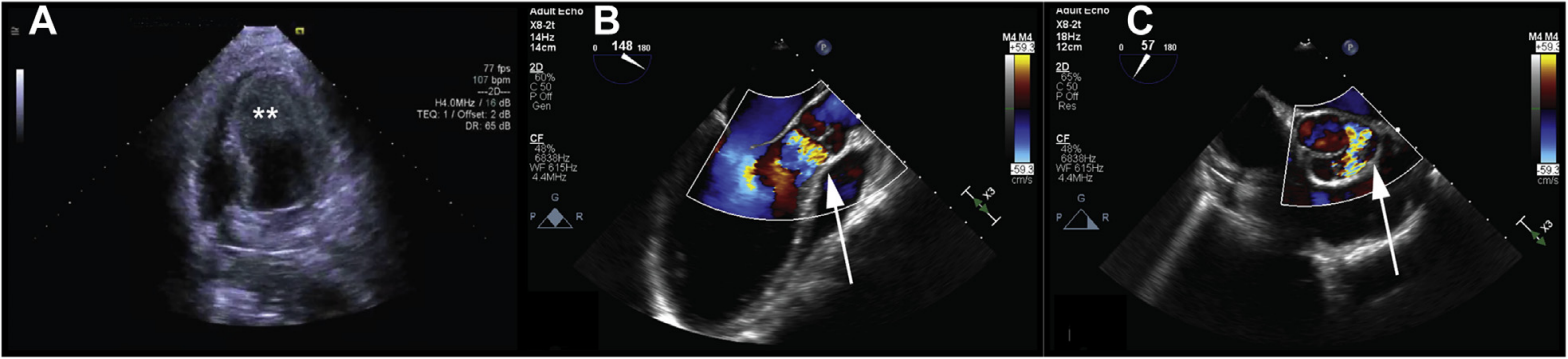
Transthoracic Echocardiography Images Transthoracic echocardiography showing **(A)** left ventricular thrombus **(asterisks)** and **(B)** and **(C)** aortic regurgitation **(arrows).**

**Figure 3:**
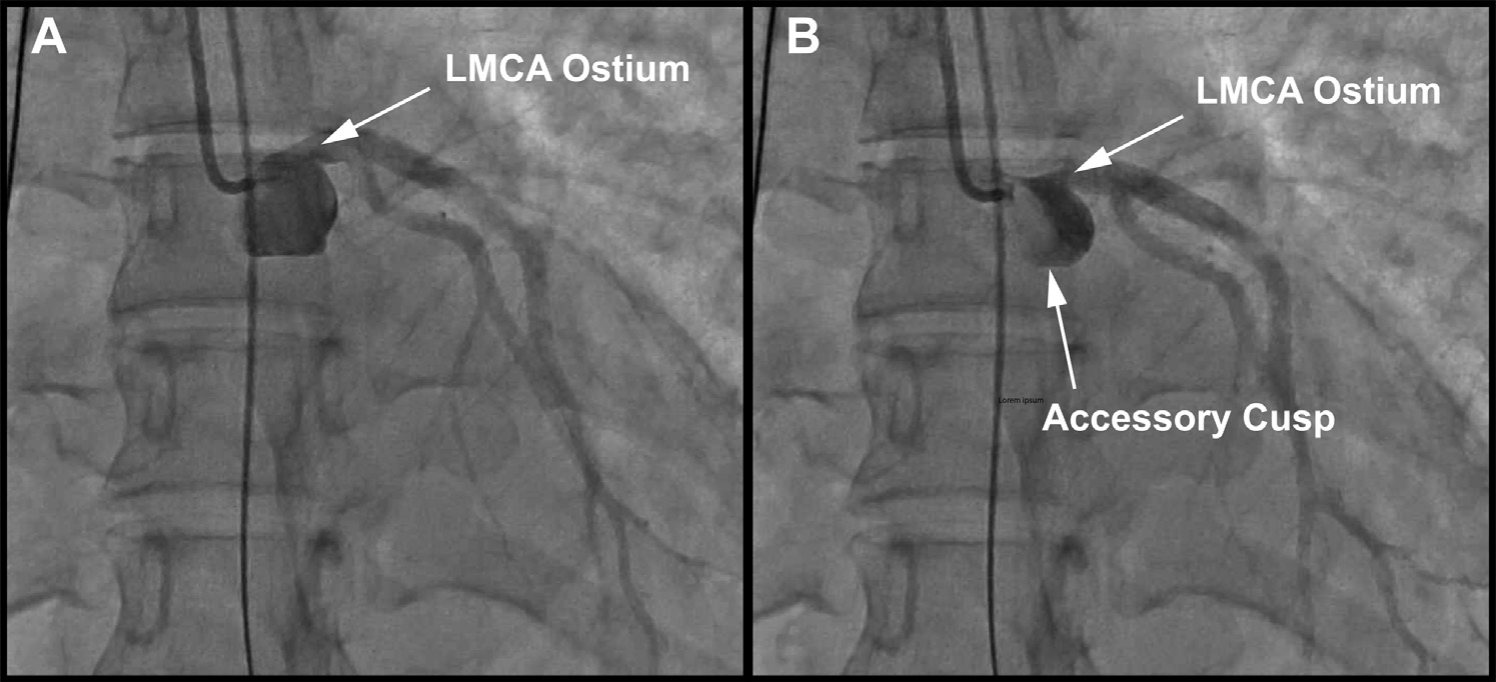
Coronary Angiogram **(A)** Imaging performed during the patient’s initial presentation. **(B)** An opacity was noted to obstruct the left main coronary artery (LMCA) intermittently during systole. This was confirmed in the operating room to be a ruptured fourth accessory cusp.

The patient had a history of congenital aortic valve disease and aortic valve insufficiency, which had been diagnosed in childhood and followed up periodically. She had been doing well until she had an episode of nausea, vomiting, diaphoresis, and chest pain while traveling for business. Evaluation in the emergency department revealed diffuse ST-segment depression on the 12-lead electrocardiogram and a mildly elevated troponin level. Transthoracic echocardiography (TTE) showed severe aortic regurgitation, a severely depressed LVEF of 5%, and LV thrombus (Figures 2A to 2C, Videos 1 and 2). Cardiogenic shock developed in the patient. Invasive angiography revealed normal coronary arteries but was suggestive of rupture of an accessory aortic valve cusp causing obstruction of the left main coronary ostium (Figures 3A and 3B, Video 3), which was confirmed intraoperatively. She underwent emergency aortic valve replacement (AVR) with a #23 Inspiris bovine tissue valve (Edwards Lifesciences). She was unable to be weaned from cardiopulmonary bypass, and post-AVR required prolonged mechanical circulatory support with central venoarterial extracorporeal membrane oxygenation with a transseptal left atrial drainage cannula for LV venting. Ten days after her initial surgery, she did not show recovery of LV function, and she underwent durable LVAD implantation as a bridge to either recovery or heart transplantation.

Over the following 6 months, the patient demonstrated myocardial recovery resulting from the combination of surgical correction of her valvular disease, relief of coronary obstruction, heart failure–guided medical therapy, and mechanical unloading of her left ventricle. TTE showed normal bioprosthetic aortic valve function with intermittent opening and an LVEF of 55%. The patient successfully passed the LVAD weaning protocol, including resting invasive hemodynamic assessment by right-sided heart cardiac catheterization and cardiopulmonary exercise stress testing with reduced LVAD support by decreasing the revolutions per minute. A nongated chest computed tomography (CT) scan did not show any bioprosthetic valve abnormalities. The team decision was then made for LVAD explantation 9 months after surgical implantation.

## Differential Diagnosis

The differential diagnoses included aortic valve dysfunction and intraoperative injury of the coronary arteries.

## Investigations

**Figure 4:**
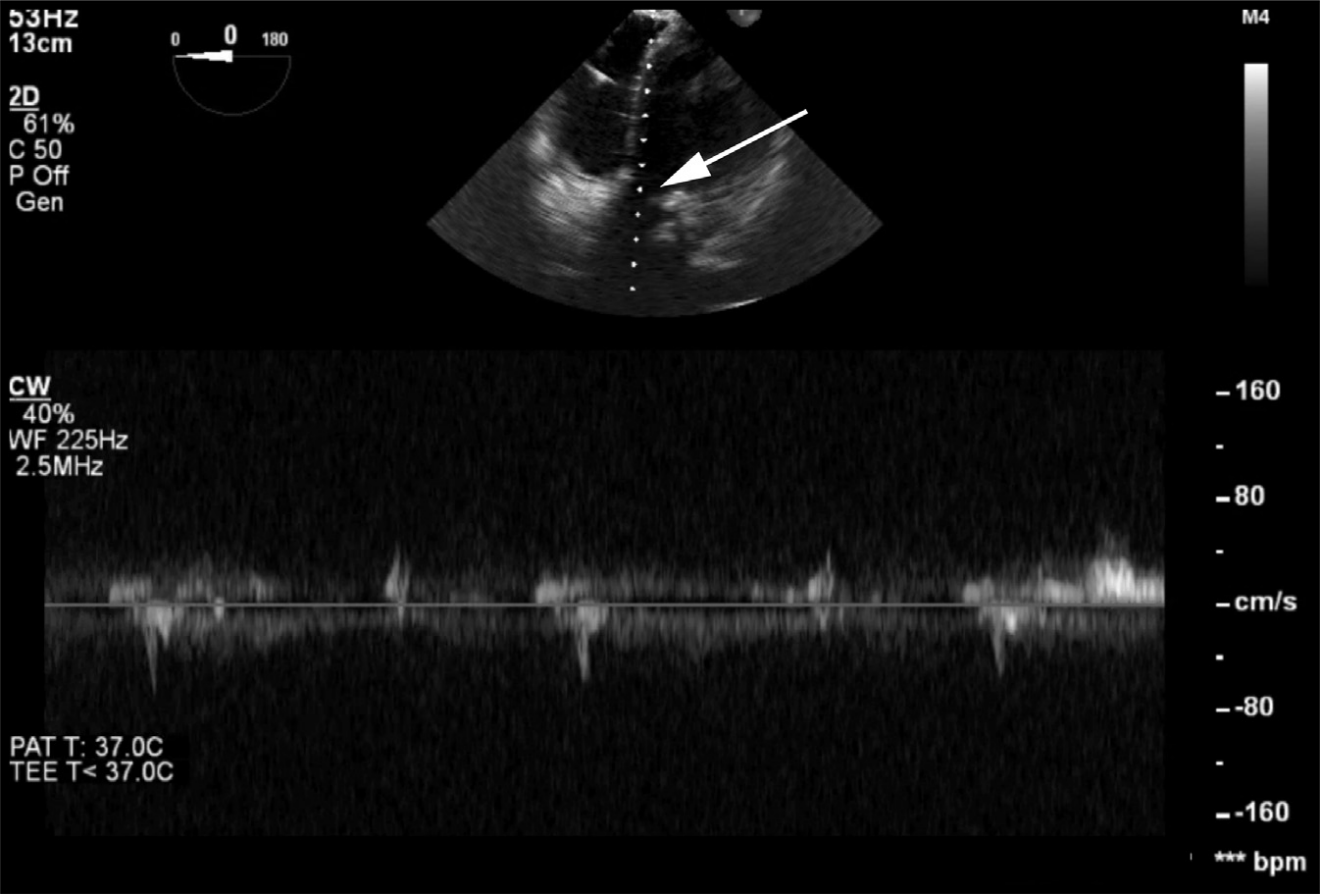
Doppler Interrogation of the Aortic Valve The image (the **arrow** denotes the line of interrogation passing through the left ventricular outflow tract) indicated no flow through the prosthesis.

Intraoperative transesophageal echocardiography (TEE) revealed no flow through the patient’s bioprosthetic aortic valve as a result of acute stenosis with fusion of the valve leaflets and subvalvular thickening as the culprit of the patient’s acute decompensation (Figure 4, Video 4).

## Management

**Figure 5:**
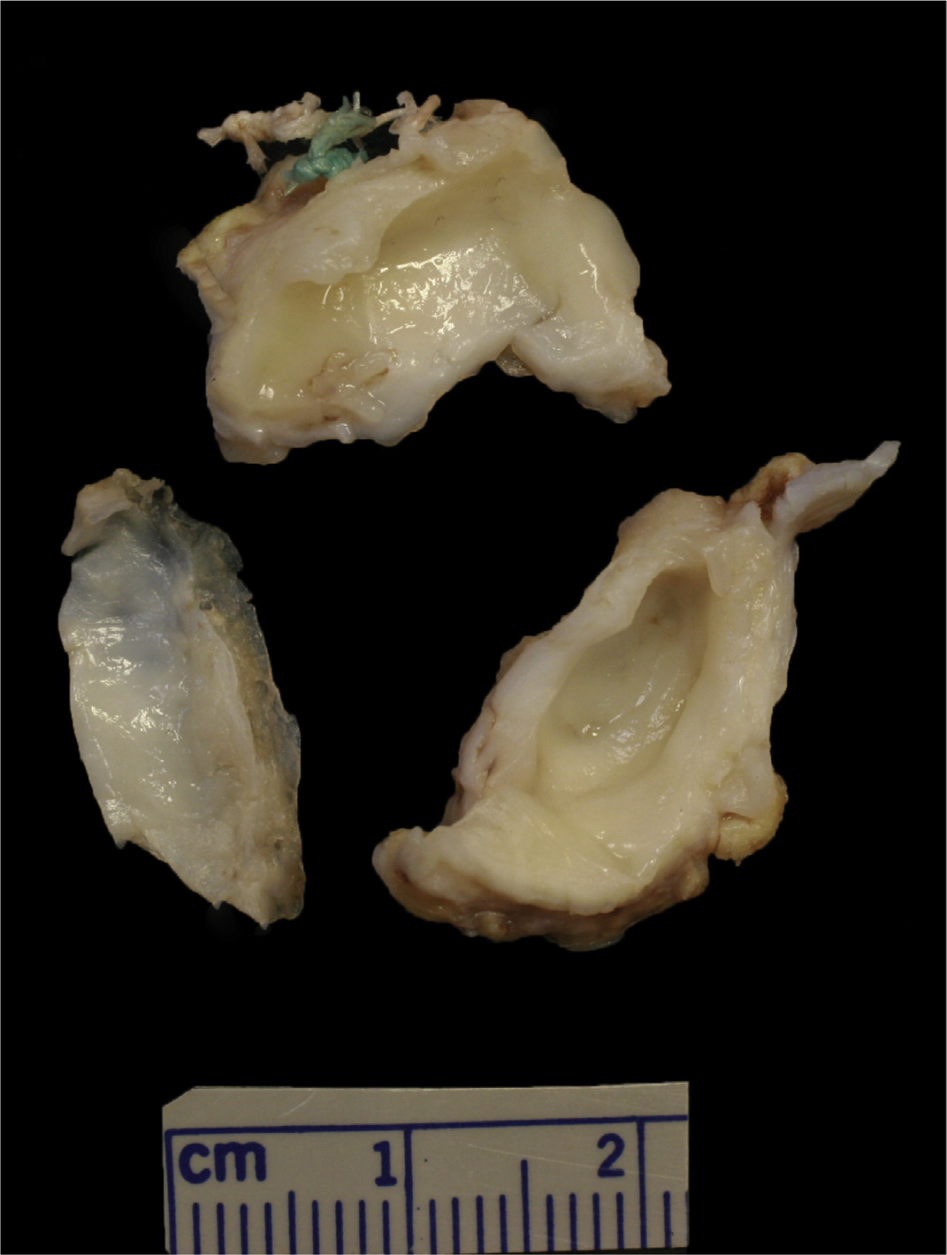
Gross Morphology of the Patient’s Excised Bioprosthetic Aortic Valve The photograph shows leaflet thickening and fibrosis Photo courtesy William C. Roberts, MD, and Saba Ilyas.

After discussion with the patient’s family, an ad hoc AVR with a #19 On-X mechanical valve (CryoLife) was performed. Excision of the previously implanted bioprosthetic valve showed a significant amount of subvalvular fibrotic tissue that was obliterating the LV outflow tract. In addition, the bioprosthetic valve leaflets themselves showed significant fibrosis and fusion (Figure 5).

Immediately after AVR, LV function and LV cavity size remained within normal limits, and the patient underwent LVAD explantation. Postoperatively, the patient did well and was discharged home.

## Discussion

At present there is no definitive way to predict accurately which patients may have LV recovery during LVAD support and which patients may go on to require heart transplantation.^1^ In general, aortic valve diseases are less significant in patients with either destination or bridge to transplantation LVAD, but they certainly may be more consequential and could affect hemodynamic stability in patients who undergo LVAD support as a bridge to recovery. In hearts with native valves, LVAD flow decreases the LV pressure and subsequently increases the pressure gradient across the aortic valve between the aorta and LV, thus effectively keeping the aortic valve closed.^2^, ^3^, ^4^

Continuous closure of the aortic valve, along with an increase in mechanical stress, may ultimately lead to deterioration and valve insufficiency or leaflet fusion. In patients with mechanical AVR, the risk of valve thrombosis and thromboembolism have precluded many patients from LVAD support; in contrast, bioprosthetic AVR (bAVR) has a much lower risk of thrombosis, and small case series suggest that outcomes in these patients are no different from the general group of LVAD recipients.^4^ LVAD recipients with bAVR require careful follow-up with attention to valve function for signs of valve degeneration, insufficiency, leaflet fusion, and thrombosis. Patients with signs of LV recovery who are being considered for explantation should undergo preoperative evaluation to determine appropriate valve type and sizing in the event a second valve replacement is needed. Periodic valve surveillance is also important in patients undergoing bridge-to-transplant therapy or destination therapy, given the long duration for which these patients often require mechanical support; on occasion, catheter or surgical intervention is required in these groups as well.

**Figure 6:**
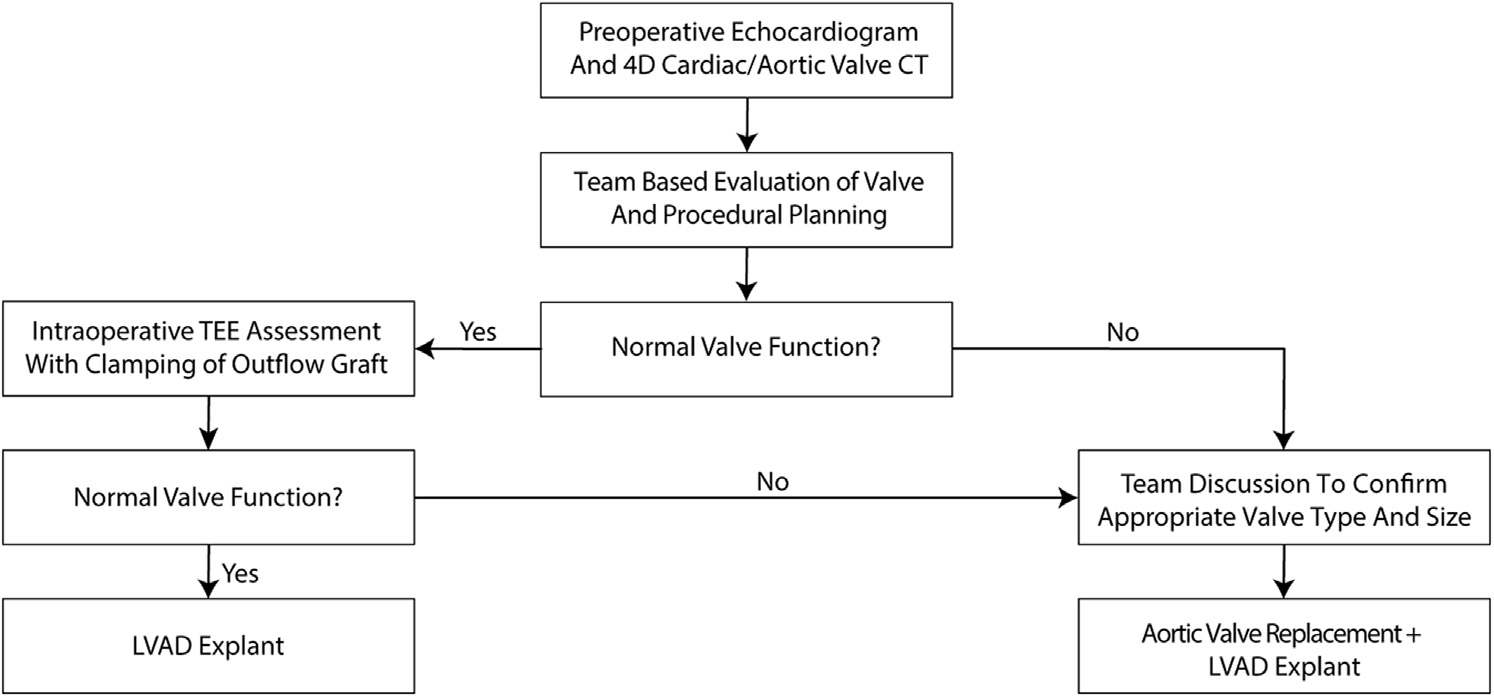
Algorithm for Evaluating Aortic Valve Function in Patients With Pre-Existing Bioprosthetic Valves Who Are Undergoing LVAD Explantation CT = computed tomography; LVAD = left ventricular assist device; TEE = transesophageal echocardiography; 4D = 4-dimensional.

Preoperatively, TTE did not suggest obvious bioprosthetic aortic valve dysfunction with intermittent opening of the prosthesis, and the decision was made to proceed with LVAD explantation. However, TTE analysis of bioprosthetic aortic valves can be difficult in LVAD patients in view of shadow artifact originated by the inflow cannula and LVAD flow, thus potentially obscuring valve anatomy and Doppler analysis. Our case demonstrates that bioprosthetic valve dysfunction can develop quickly, and as such, intraoperative evaluation should also be performed at the time of explantation. We have developed an algorithm that can be followed in preoperative planning (Figure 6). We recommend that patients with a bioprosthetic aortic valve and an LVAD who are undergoing explantation should ideally have preoperative TTE and gated retrospective cardiac 4-dimensional (4D) CT scan with a focus on bioprosthetic aortic valve anatomy and function. Although our patient had a nongated chest CT scan, gated retrospective 4D CT can provide more detailed assessment of valvular function throughout both systole and diastole when echocardiography does not provide adequate 2-dimensional imaging of the valve. Moreover, physiological assessment of valve gradients is difficult in patients with an LVAD with TTE because there are no established data on valve gradients in this group. Moreover, if valve degeneration occurs in the interim period between outpatient evaluation and surgery (as in our case), detailed anatomical information is available to ensure appropriate valve sizing. Preoperatively, a multidisciplinary team consisting of cardiac imagers, cardiac surgeons, and heart failure specialists should discuss options for valve replacement should the unexpected need arise, including valve type and sizing. In patients with normal preoperative evaluation, we recommend TEE evaluation with a “clamp then observe” approach to ensure that no valve dysfunction has occurred in the interim period. In our case, a mechanical valve was chosen, given the young age of the patient and the desire to avoid further cardiac surgical procedures because of valve degeneration.

## Follow-Up

The patient was discharged home on postoperative day 5. She has been observed closely as an outpatient and continues to do well over 1 year after LVAD explantation.

## Conclusions

This case highlights the complexities of altered physiology in patients with LVADs and the effect it can have on bioprosthetic aortic valves. The surgical complexities of LVAD explantation necessitate evaluation of previous bioprosthetic aortic valves to ensure adequate function. Indeed, bioprosthetic valve dysfunction can develop in a relatively short time; despite adequate preoperative planning, careful intraoperative valve assessment should be performed before outflow graft clamping and LVAD explantation.

## Funding Support and Author Disclosures

The authors have reported that they have no relationships relevant to the contents of this paper to disclose.

## Appendix

Supplemental Video 1 TTE Images Showing Significant Aortic Regurgitation via Color Flow Doppler Interrogation of the Aortic Valve

Supplemental Video 2 TTE Images Showing an Apical 4-Chamber View With Severely Reduced LVEF and LV Thrombus

Supplemental Video 3 Angiography Demonstrating a Difficult to Engage Left Main Coronary Artery Resulting From the Accessory Valve Cusp With Intermittent Obstruction of the Coronary Ostium

Supplemental Video 4 Intraoperative TEE with Doppler Interrogation of the Aortic Valve Showing No Flow Through the Prosthesis
